# Identification and validation of feature genes associated with M1 macrophages in preeclampsia

**DOI:** 10.18632/aging.205264

**Published:** 2023-12-01

**Authors:** Panpan Hu, Shanshun Luo, Guangjin Qu, Qiqi Luo, Yu Tian, Kun Huang, Tingting Sun

**Affiliations:** 1 Department of Gerontology, The First Affiliated Hospital of Harbin Medical University, Harbin 150001, China

**Keywords:** preeclampsia, M1 macrophages, biomarkers, bioinformatics, multi-omics

## Abstract

Preeclampsia (PE) is a pregnancy-specific cardiovascular complication that is the leading cause of maternal and neonatal morbidity and mortality. Previous studies have indicated the importance of immune cells, such as M1 and M2 macrophages, in the pathogenesis of PE. However, the mechanisms leading to immune dysregulation are unclear. Data-independent acquisition proteomic analysis was performed on placental tissues collected from patients with PE and healthy controls. Transcriptome data for placenta samples from patients with PE and their corresponding controls were obtained from the Gene Expression Omnibus database. Differential analysis of transcriptome and proteome data between PE and control groups was performed using R software. Immunocytic infiltration scoring was performed using the quantiseq algorithm. Weighted gene co-expression network analysis (WGCNA) screened for feature genes associated with M1 cell infiltration. Protein-protein interaction (PPI) analysis identified hub genes. We confirm that the infiltration score of M1 macrophages was significantly increased in the placental tissues of patients with PE. Differential analysis, WGCNA, and PPI analysis identified four hub molecules associated with M1 cell infiltration (HTRA4, POGK, MFAP5, and INHBA). The hub molecules displayed dysregulated expression in PE tissues. The qPCR, Western blots, and immunohistochemistry analyses confirmed that Inhibin, beta A (INHBA) was highly expressed in placental tissues of patients with PE. Immunofluorescence revealed the extensive infiltration of M1 macrophages in the placental tissues of patients with PE and their co-localization with INHBA. The collective results identified hub genes associated with M1 macrophage infiltration, providing potential targets for the pathogenesis and treatment of PE.

## INTRODUCTION

Pregnancy-specific cardiovascular complication, known as Preeclampsia (PE), is characterized by hypertension and proteinuria after 20 weeks of gestation and affecting around 2% to 8% of pregnancies. PE significantly contributes to maternal and infant morbidity and mortality [1]. Despite extensive research into the cause of PE over recent decades, its pathogenesis remains unclear. The current diagnostic method for PE involves identifying hypertension and proteinuria, which lacks sensitivity and specificity and results in poor outcomes for both mothers and infants [2]. Therefore, early assessment of high-risk pregnant women is crucial while accurately identifying, diagnosing, and treating PE patients is essential for reducing adverse maternal and fetal outcomes and improving neonatal and maternal outcomes.

In recent years, early diagnostic/prognostic biomarkers have emerged as a prominent research topic, with several studies focusing on biomarkers associated with PE. The placenta’s role in the pathogenesis of PE is significant, and shallow placental invasion is a crucial factor in its development [3]. According to the immune dysfunction theory, maintaining the ratio of M1 and M2 macrophages is critical for a healthy pregnancy, where the decidua progresses from an M1-dominant phenotype during implantation to an M2-dominant phenotype during embryonic development. However, in patients with preeclampsia, this ratio becomes imbalanced with overexpression of M1 macrophages, indicating alterations in maternal inflammatory status [4]. Nevertheless, the precise molecular mechanism that leads to shallow placental invasion remains unresolved [5].

In recent years, proteomic and bioinformatic analyses have been used to identify differentially expressed proteins, transcriptome changes, and their physiological functions in many diseases. These advancements have improved understanding of the pathophysiological processes of these diseases [6]. Currently, some bioinformatics methods are being used for large-scale gene expression profile studies to screen key differentially expressed genes between PE placenta and age-matched controls, as well as PE-related proteins.

The aim of this study was to integrate differential expression and identify target genes closely related to immune infiltration, to further explore disease-related factors and identify feasible biomarkers. Our results could provide further understanding of the molecular mechanisms of PE and will help inform the selection of appropriate targets for effective treatment.

## MATERIALS AND METHODS

### Clinical case collection and sample collection

The research included eight patients diagnosed with PE and four patients with normal pregnancies who underwent cesarean section at the First Affiliated Hospital of Harbin Medical University (Harbin, China) between September 2021 and September 2022. Those with diabetes, metabolic syndrome, kidney disease, infections, chromosomal abnormalities, or structural/congenital malformations were excluded from the study. Please refer to Supplementary Table 1 for detailed clinical data on the participants. Placental tissue samples were collected immediately after delivery from the area surrounding the parturient woman while avoiding vessels and calcification and stored at −80°C until analysis. The study adhered to the principles outlined in the Helsinki Declaration and was approved by the Research Ethics Committee of the First Affiliated Hospital of Harbin Medical University (No. 2022177). After being informed of the nature of the study, all participants provided written informed consent.

### Data-independent acquisition (DIA) proteomics analysis

DIA proteomics technology was used to conduct proteomic analysis on eight samples of PE and four normal samples. Q-Exactive HF liquid chromatography-mass spectrometry (Thermo Fisher Scientific, San Jose, CA, USA) was employed to generate precise and highly reproducible mass spectrometry quantitative data analysis of many proteins [7]. Protein expression changes in ratio values and *P*-values were identified and quantified among 12 samples using the mProphet algorithm based on the spectral library constructed using traditional Data-dependent Acquisition mode. Credible protein expression levels were used in principal component analysis (PCA) to investigate the relationship between diverse sample dimensions. Two benchmarks were employed to estimate the differences among samples using credible proteins. Fold-change was used to assess changes in protein expression levels, and *t*-tests were utilized to determine the significance of the differences between samples. For this project, differential screening criteria were established as having a fold-change of ≥1.5 and *P* < 0.05.

### Transcriptome data collection and differential analysis

The transcriptome sequencing data for 17 PE patient placental tissues and 26 control normal placental tissues were obtained from the Gene Expression Omnibus database (https://www.ncbi.nlm.nih.gov/geo/), and the dataset with data number GSE10588 was selected. The differential analysis was performed using the “Limma” package in R software [8]. Differentially expressed genes were screened with a threshold of having a fold-change ≥1.5 and *P* < 0.05.

### Enrichment analysis

Gene Ontology (GO) is a commonly used method for analyzing gene big data, which includes three categories: Biological Process, Cellular Component, and Molecular Function [9]. Kyoto Encyclopedia of Genes and Genomes (KEGG) and Reactome were utilized to explore signaling pathways associated with gene sets. ClusterProfiler package in R software was employed in conducting enrichment analyses based on GO, KEGG, and Reactome. Significant enrichment was identified by a *P*-value below 0.05 and a False Discovery Rate (FDR) below 0.1.

### Immune infiltration analysis

Transcriptome data was utilized for immune infiltration analysis, which was conducted using the immunedeconv package that supports several algorithms for immune infiltration analysis. In this study, the quantiseq algorithm was employed to assess the infiltration scores of 10 different immune cells in both the placental tissues of 17 PE patients and 26 normal control placental tissues. The 10 immune cells include B cell, Macrophage M1, Macrophage M2, Monocyte, Myeloid dendritic cell, Neutrophil, NK cell, T cell CD4+ (non-regulatory), T cell CD8+, and T cell regulatory (Tregs).

### Weighted gene co-expression network analysis (WGCNA)

WGCNA is a systems biology approach that describes gene correlation patterns between different samples. It is used to identify gene sets with highly coordinated changes and to identify potential biomarker genes or therapeutic targets based on the connectivity within gene sets and their association with phenotypes. In this study, we used the WGCNA method to screen for core genes associated with M1 macrophage infiltration. To create a co-expression network, the WGCNA package in R software was utilized. Initially, sample clustering was conducted for identifying potential outliers. Afterwards, an automated network construction function was employed to construct a co-expression network. The pickSoftThreshold R function was utilized to determine the soft threshold power β and suggest co-expression similarity for calculating adjacency. Hierarchical clustering and dynamic tree cut functions were further used to detect modules. Finally, gene significance and module membership were evaluated, and the module was linked to M1 macrophage content. Relevant information about the module genes was extracted to facilitate supplementary analysis.

### Protein-Protein Interaction (PPI) network

*GeneMANIA*, which can be accessed through http://genemania.org, is a user-friendly online database that simplifies the investigation of gene or gene set functionality and interactions [10]. With data on 166,691 genes from nine species and a vast collection of 660,554,66 interactions, *GeneMANIA* provides researchers with valuable information. In this study, protein interaction data related to target proteins in human participants was employed via *GeneMANIA* to build a PPI network [11].

### Transcription factor-microRNA (TF-miRNA)-target regulated network and drug-target regulated network

The Networkanalyst (https://www.networkanalyst.ca/NetworkAnalyst/home.xhtml) online tool was utilized in this study to investigate the regulatory networks of core genes by analyzing transcription factors and miRNAs [12]. In addition, data on the associations between mono-components of Traditional Chinese Medicine and their respective targets were acquired through the HERB database (http://herb.ac.cn/) [13]. This information was then presented visually using Cytoscape software to facilitate understanding of the complex relationships involved.

### Western blotting

Tissue samples were prepared by cutting and grinding before being lysed with ice-cold RIPA buffer containing 1% protease inhibitors for 30 minutes. The total protein content was collected from the supernatant after centrifugation at 13,500 rpm for 15 minutes at 4°C. Protein concentration was determined using the bicinchoninic acid method. Subsequently, an equivalent amount of total protein was separated through sodium dodecyl sulfate-polyacrylamide gel electrophoresis (SDS-PAGE) set at 8–12%, followed by transfer onto a polyvinylidene fluoride membrane. After blocking the membrane with 5% skimmed milk for 2 hours, it was incubated overnight at 4°C with primary INHBA antibody (PTG, Chicago, IL, USA). A secondary fluorescent antibody (LI-COR Biosciences, Lincoln, NB, USA) was then used to bind to the primary antibody at room temperature for 1 hour. Employing the Odyssey CLx imaging system (LI-COR Biosciences), the changes in protein levels relevant to these groups were evaluated, where glyceraldehyde 3-phosphate dehydrogenase (GAPDH) was employed as an internal control. Lastly, Image Studio software (LI-COR Biosciences) was utilized to analyze and process the protein bands detected.

### Real time-quantitative polymerase chain reaction (RT-qPCR)

Total RNA was extracted from cells using an RNA extraction kit (Axygen Scientific, Union City, CA, USA). Subsequently, cDNA was synthesized using a reverse transcription kit (Toyobo, Osaka, Japan). The gene expression was detected through SYBR Green dye (Thermo Fisher Scientific, Waltham, MA, USA), where GAPDH was employed as the internal reference gene. Data analysis was conducted utilizing the 2^−ΔΔCt^ method. The primers used for amplification were: Inhibin, beta A (INHBA) forward primer: GTCCTTCCACTCAACAGTCATC, reverse primer: GTACAACATGGACATGGGTCTC; GAPDH forward primer: CTGGGCTACACTGAGCACC, reverse primer: AAGTGGTCGTTGAGGGCAATG.

### Immunofluorescence

Initially, human chorionic membrane sections are subjected to a heating process at 60°C for one hour. After that, the slices are deparaffinized with ethanol and rehydrated using heavy water. To decrease non-specific antigen responses and endogenous peroxidase activity, segments are treated with a solution containing 1% H_2_O_2_. Then, the anti-INHBA antibody (PTG, Chicago, IL, USA) and CD86 antibody (PTG, Chicago, IL, USA) are incubated at 4°C for 20 hours to facilitate fluorescence staining. The resulting images are captured under uniform light intensity conditions using a fluorescence microscope (DM4B, Leica, Wetzlar, Germany). Subsequently, ImageJ software (NIH) is employed to quantitatively analyze the fluorescence intensity by adjusting threshold values before comparative analysis to the control group. For localization analysis of the human chorionic tissue, all cell spots in each image are inspected and analyzed through utilization of the JACoP plugin found in the ImageJ software (NIH, Bethesda, MD, USA).

### Statistical analyses

When comparing continuous variables between two groups, either a *t*-test or Mann-Whitney *U* test was conducted depending on the normality distribution of the data. Spearman’s correlation analysis was employed for all correlation analyses. Statistical analysis for this study was executed using R software. Significance was determined when *P*-value was less than 0.05 (^*^P < 0.05; ^**^P < 0.01; ^***^P < 0.001 based on the experiment).

### Data availability statement

The original data generated from this study can be found in the Supplementary Materials. The identification number of the public data used in this study has been described in the methodology section.

## RESULTS

### DIA protein analysis and differential proteins between PE and normal placental tissues

**Figure 1:**
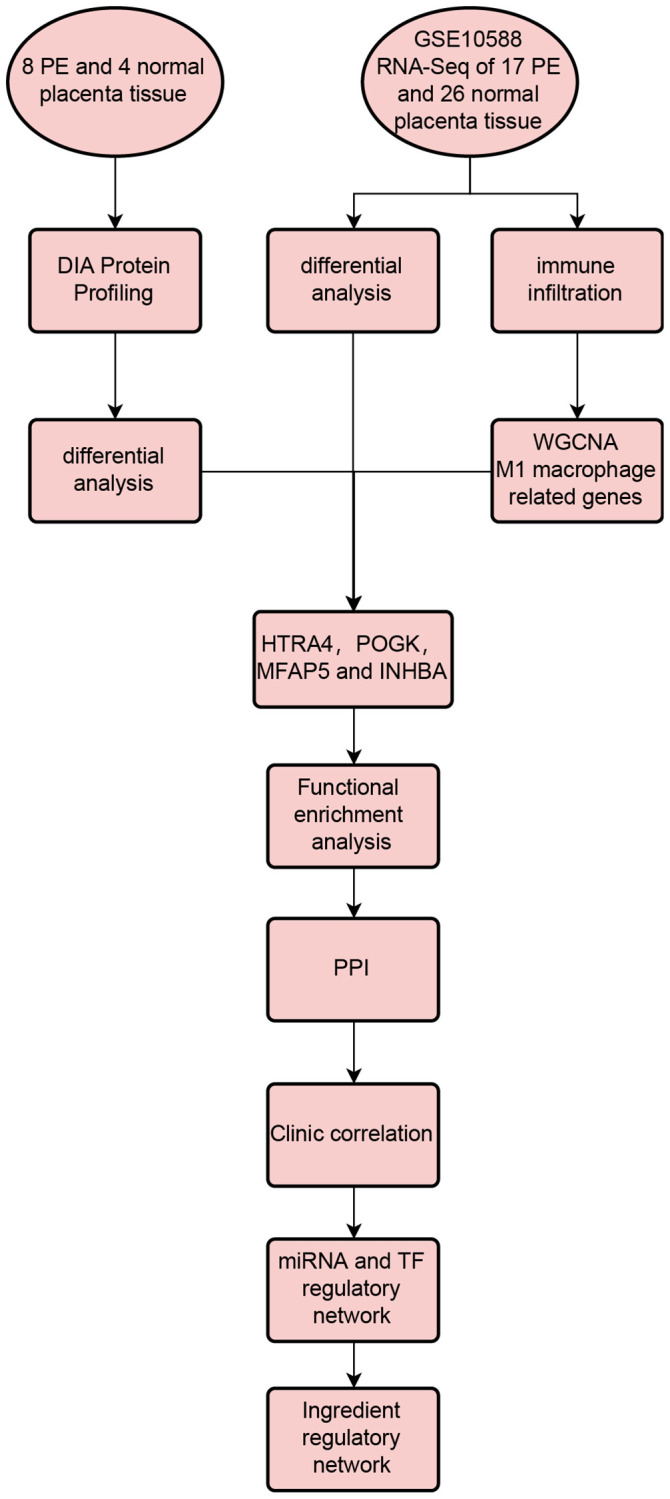
Workflow of this study.

**Figure 2:**
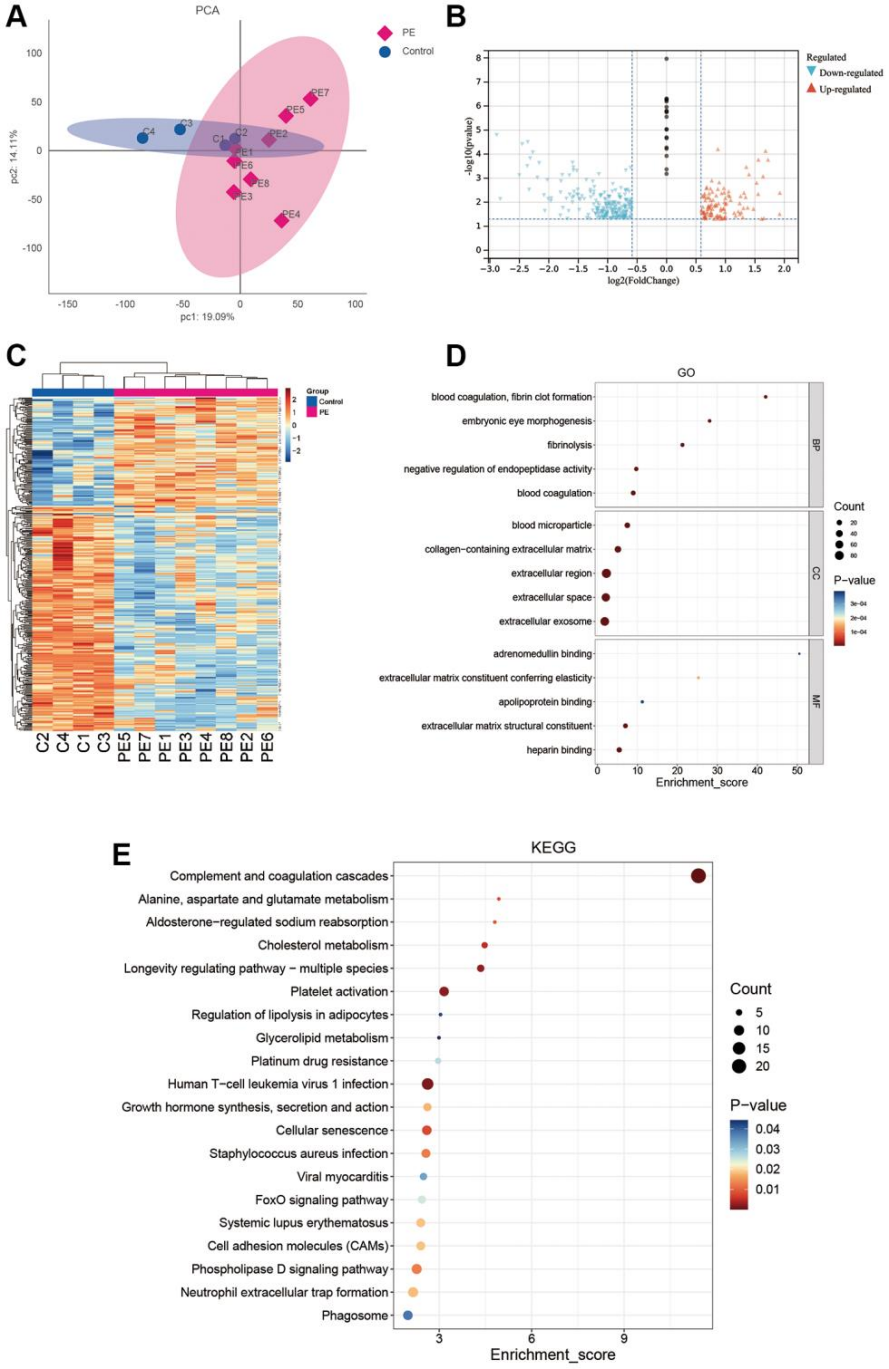
**DIA proteomics and functional enrichment analyses.** (**A**) PCA analysis of clinical samples. (**B**–**E**) Volcano plot (**B**), heat map (**C**), GO enrichment analysis (**D**), and KEGG enrichment analysis (**E**) of differentially expressed proteins between the PE group and normal controls.

Figure 1 illustrates the experimental flowchart. PCA demonstrated that PE tissues and normal tissues could be well-distinguished (Figure 2A). Differential analysis revealed that compared to normal placental tissues, 216 proteins were downregulated and 106 proteins were upregulated in PE tissues (Figure 2B, 2C). Supplementary Table 2 shows the expression matrix of each protein in both PE and control placental tissues. Through GO analysis, it was discovered that these proteins are linked to a multitude of biological processes such as fibrinolysis, embryonic eye morphogenesis, blood coagulation, negative regulation of endopeptidase activity and fibrin clot formation. These proteins are primarily situated in regions including extracellular exosome, extracellular space, collagen-containing extracellular matrix, blood microparticles, and extracellular region (Figure 2D). Additionally, according to KEGG analysis, these proteins partake mainly in pathways such as the FOXO signaling pathway, cell adhesion molecules, Phospholipase D signaling pathway, neutrophil extracellular trap formation, and phagosome (Figure 2E).

### Differential mRNA and immune infiltration between PE and normal placental tissues

**Figure 3:**
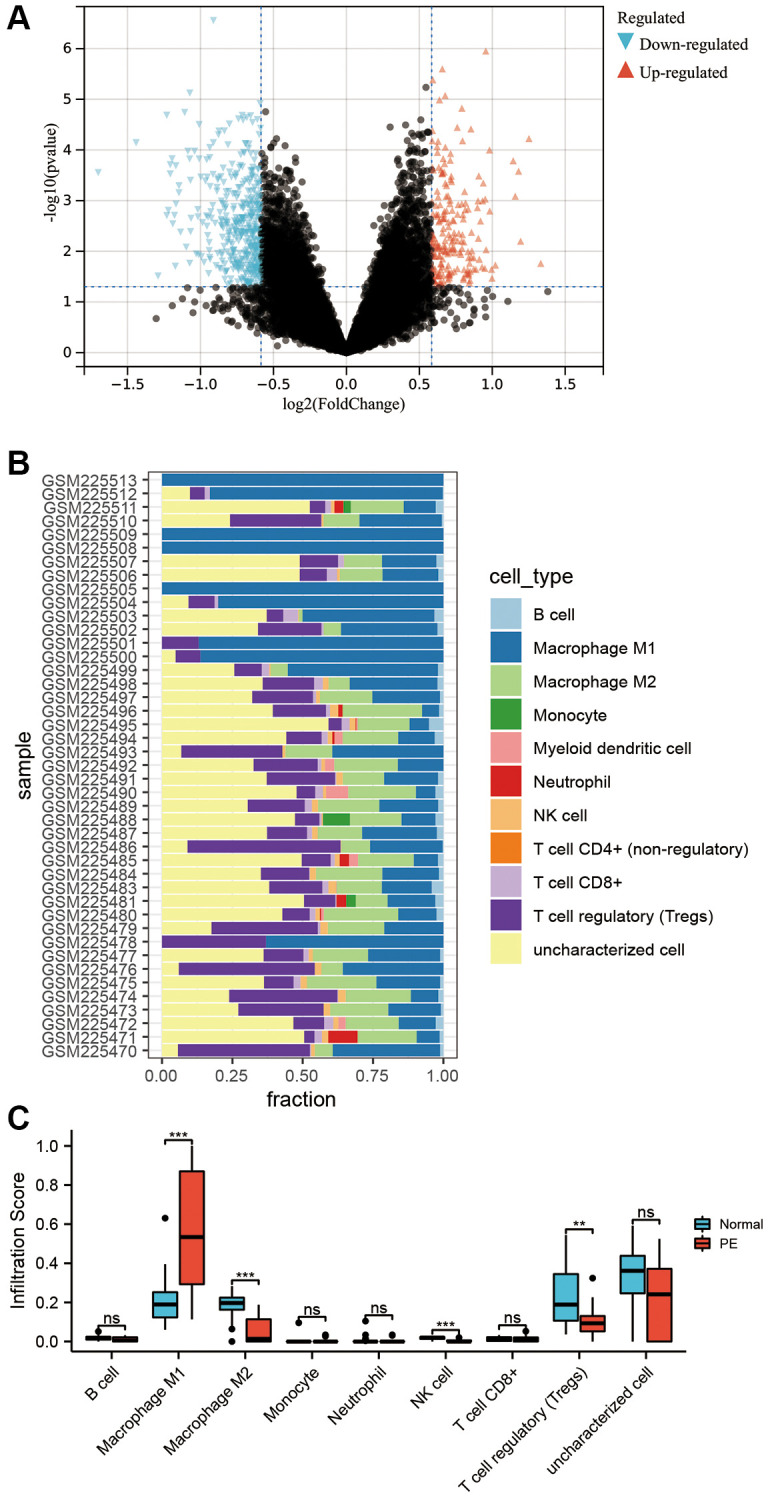
**Transcriptome differential analysis and immune infiltration.** (**A**) Volcano plot of differentially expressed mRNA in the GSE10588 dataset between the PE group and normal controls. (**B**) Scores of 10 types of immune cell infiltration in each sample of the GSE10588 dataset. (**C**) Differences in immune cell infiltration scores between the PE group and normal controls in the GSE10588 dataset.

By performing a differential analysis of GSE10588, it was determined that there were 864 upregulated mRNAs and 727 downregulated mRNAs in PE tissues when compared to normal placental tissues (Figure 3A). Following this, infiltration scores for 10 different types of immune cells were calculated for each sample in GSE10588 (Figure 3B). Comparing the results to those of normal placental tissues, M1 macrophages were observed to be significantly increased while the levels of M2 macrophages, natural killer (NK) cells, and T regulatory (Treg) cells were significantly decreased in PE placental tissues (Figure 3C).

### WGCNA

**Figure 4:**
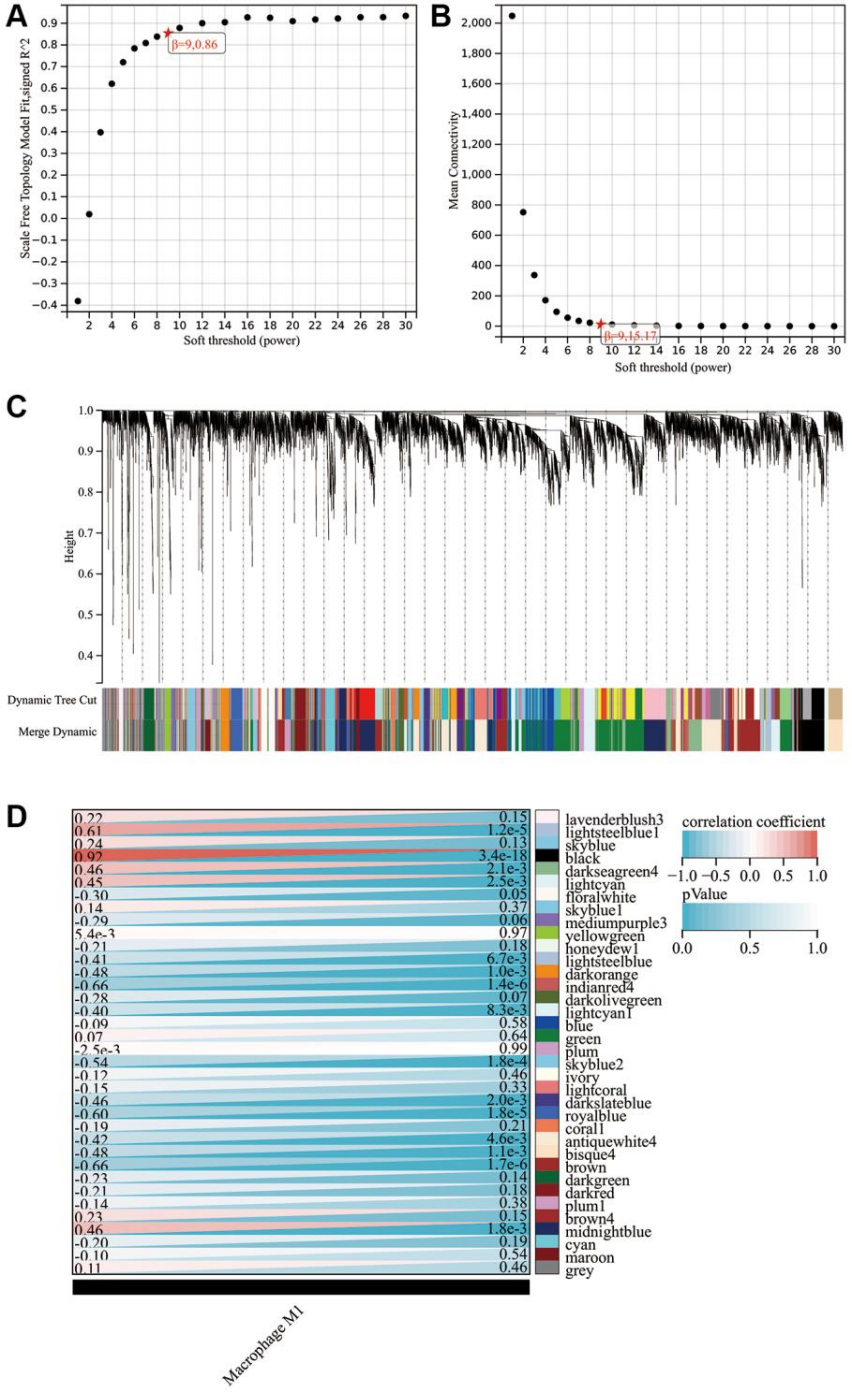
**WGCNA findings.** (**A**) Scale independence plot. (**B**) Mean connectivity plot. (**C**) Gene clustering dendrogram. (**D**) Heat map showing the correlation between modules and M1 macrophages.

Given our finding that M1 macrophages were significantly upregulated in PE placental tissues and contributed to the occurrence and development of PE, we attempted to screen for mRNA associated with M1 macrophage infiltration through WGCNA. The optimal soft threshold was β = 9 (Figure 4A, 4B). Under this soft threshold, genes in the GSE10588 dataset were grouped into 36 modules (Figure 4C, 4D). The correlation between 36 modules and the infiltration of M1 macrophages was analyzed, indicating that black, midnightblue, and darkseagreen4 modules had a strong positive relationship with M1 macrophage infiltration. Correlation coefficients were measured at 0.92, 0.46, and 0.46, respectively (Figure 4D). However, lightsteelblue1 and lightcyan modules had significant negative correlations with M1 macrophage infiltration with correlation coefficients reaching −0.41 and −0.4, respectively (Figure 4D). A total of 378 mRNAs were included in further analyses from these five modules.

### Screening of hub proteins associated with M1 macrophage infiltration in PE

**Figure 5:**
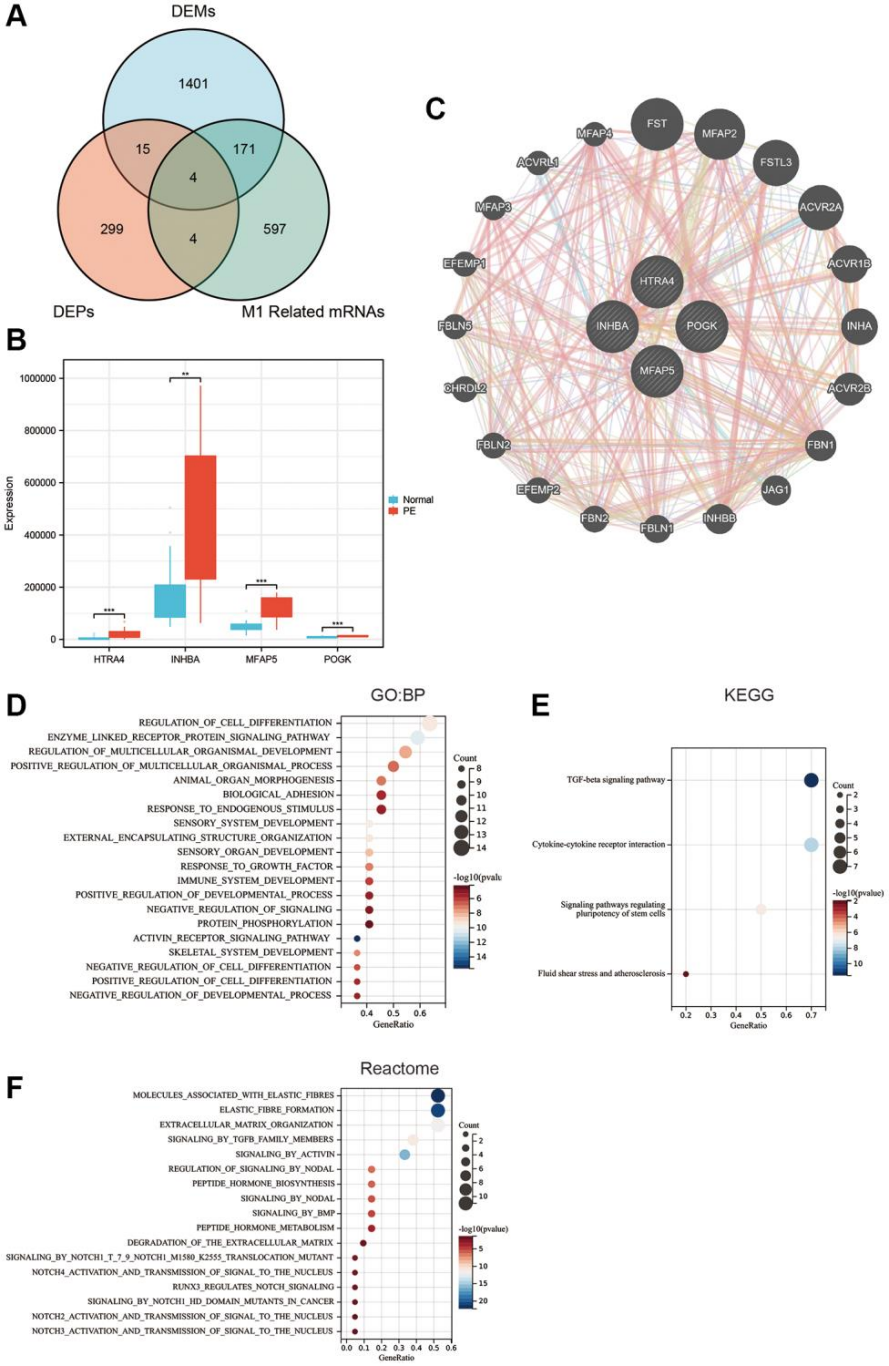
**Hub protein screening and enrichment analysis.** (**A**) Venn diagram of the intersection of differentially expressed proteins, differentially expressed mRNA, and M1 macrophage-related mRNA. (**B**) Box plot of differentially expressed hub proteins. (**C**) Protein-protein interaction network. (**D**) GO enrichment analysis. (**E**) KEGG enrichment analysis. (**F**) Reactome enrichment analysis.

To obtain proteins that were dysregulated in PE and correlated with M1 macrophage infiltration, determination of the intersection of differential proteins, differential mRNAs, and M1 macrophage infiltration-related mRNAs revealed four proteins (Figure 5A): HTRA4, POGK, MFAP5, and INHBA, which all highly expressed in the PE tissue (Figure 5B). All four proteins were upregulated in PE tissues. Figure 5C displays the top 20 proteins closely interacting with HTRA4, POGK, MFAP5, and INHBA. GO analysis showed that these proteins in the PPI network were associated with biological processes, such as regulation of cell differentiation, enzyme-linked receptor protein signaling pathway, regulation of multicellular organismal development, positive regulation of multicellular organismal process, and others (Figure 5D). The KEGG analysis demonstrated that the proteins had associations with diverse signaling pathways, comprising transforming growth factor-beta (TGFβ), cell-cell cytokine receptor interaction, pluripotency of stem cells regulation signaling pathways, fluid shear stress, atherosclerosis, among other pathways (Figure 5E). The Reactome analysis depicted the proteins’ involvement in multiple signaling pathways involving molecules that are associated with elastic fibers, formation of elastic fibers, extracellular matrix organization, signaling by TGFβ family members, activation through Activin, and more (Figure 5F).

### Association of hub proteins with clinical features of PE

**Figure 6:**
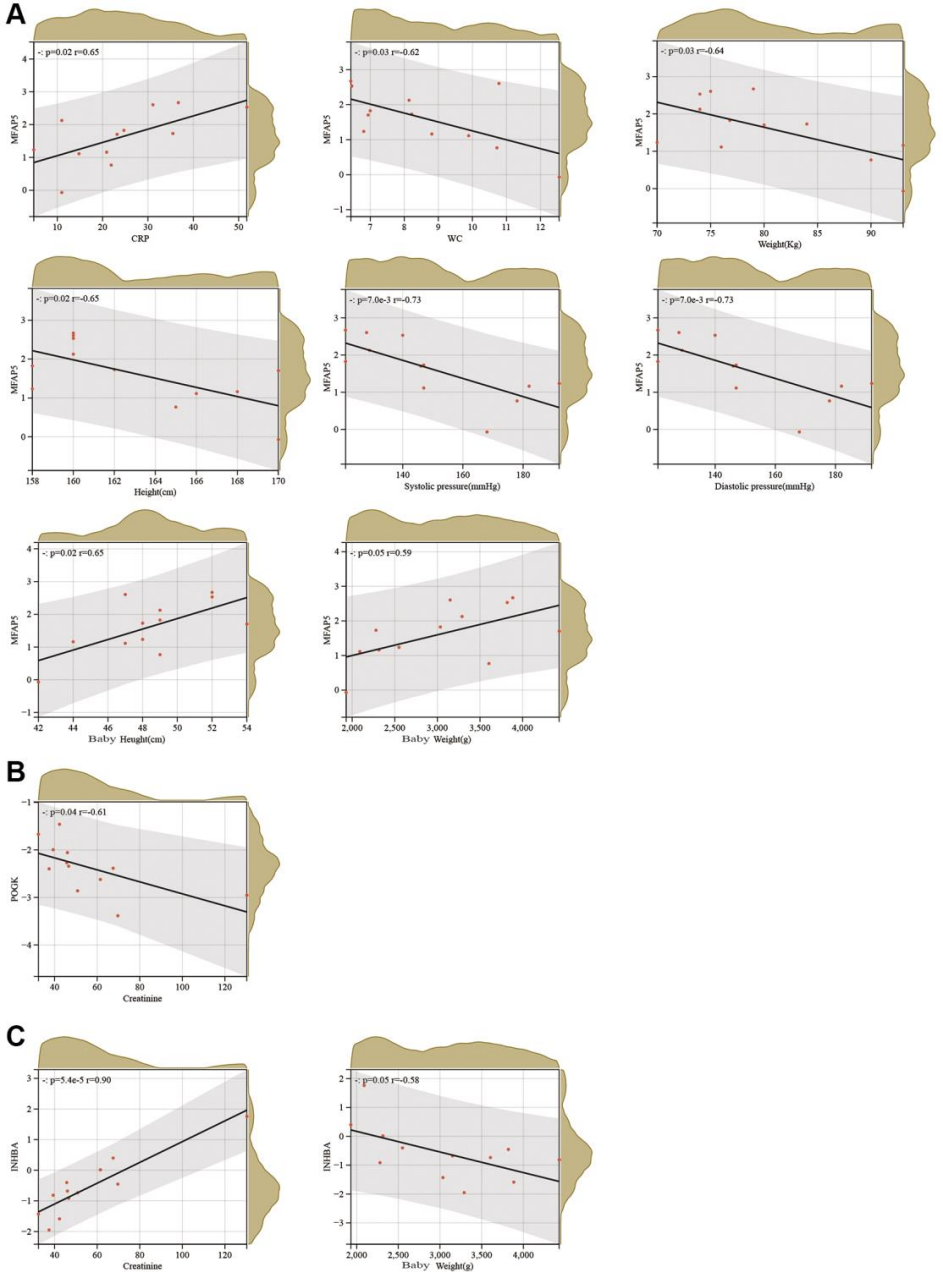
**Correlation analysis between hub proteins and clinical characteristics of PE.** (**A**–**C**) Correlation analysis between MFAP5 (**A**), POGK (**B**), and INHBA (**C**) and clinical characteristics of PE.

We carried out a correlation analysis combining HTRA4, POGK, MFAP5 and INHBA protein expression levels with relevant clinical features in patients affected by PE. Our findings revealed that MFAP5 exhibited a positive correlation with CRP, newborn weight, and newborn height; whereas the protein was negatively associated with white blood cells, maternal pre-pregnancy weight, maternal height, systolic blood pressure, and diastolic blood pressure (as depicted in Figure 6A). On the other hand, POGK had a negative connection with creatinine levels (as demonstrated in Figure 6B), while INHBA showed a positive interface with creatinine levels but had a striking negative association with newborn weight (as demonstrated in Figure 6C).

### Regulatory network of hub proteins

**Figure 7:**
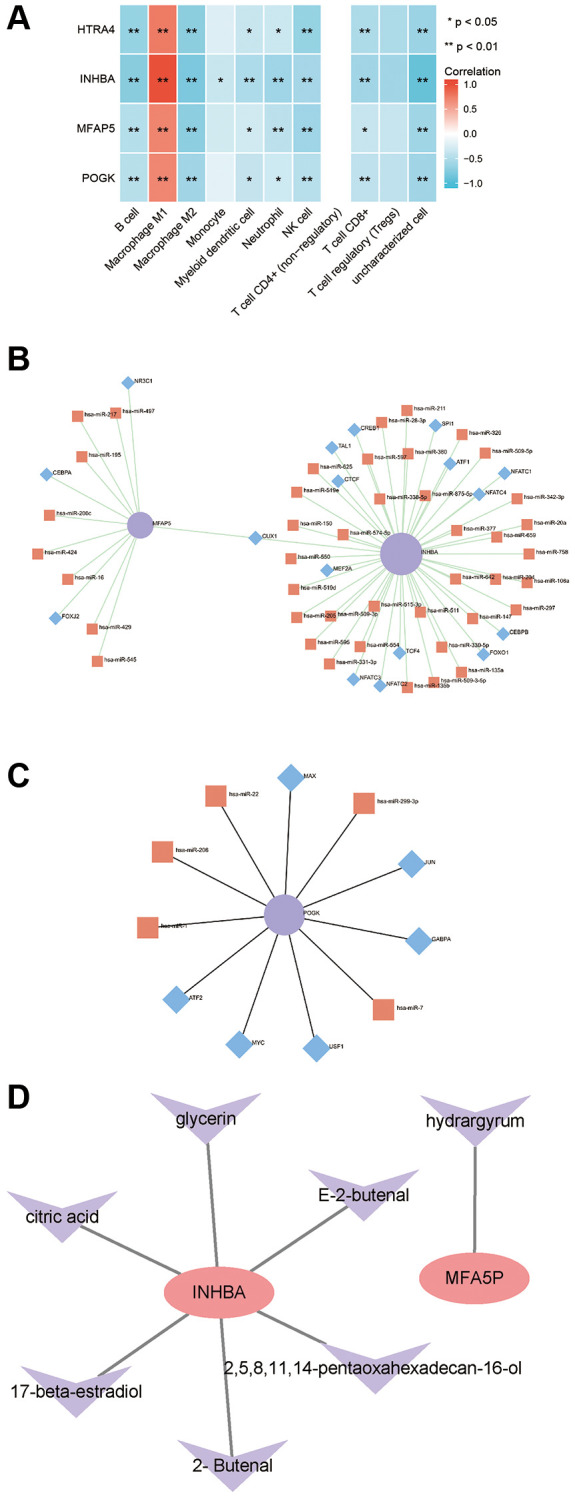
**Correlation between hub proteins and immune infiltration and regulatory network.** (**A**) Correlation analysis of hub genes with immune infiltration. (**B**) miRNA/TF regulatory network of MFAP5 and INHBA. (**C**) miRNA/TF regulatory network of POGK. (**D**) Traditional Chinese medicine monomer-target protein regulatory network of MFAP5 and INHBA.

The correlation analysis showed that the mRNA expression levels of High temperature requirement A4 (HTRA4), Pogo transposable element derived with KRAB domain (POGK), Microfibrillar-associated protein 5 (MFAP5), and INHBA were positively correlated with M1 macrophage infiltration score at the transcription level, while negatively correlated with B cells, M2 macrophages, monocytes, dendritic cells, neutrophils, NK cells, CD8+ T cells, and Treg cell infiltration scores (Figure 7A). To explore potential factors that may regulate HTRA4, POGK, MFAP5, and INHBA expression, we predicted their miRNAs and TFs through the online tool Networkanalyst. The molecules collected by Networkanalyst included MFAP5, INHBA, and POGK. Figure 7B shows the miRNAs and TFs that regulate them; CUX1 simultaneously regulated the expression of MFAP5 and INHBA. Figure 7C shows the miRNAs and TFs that regulate POGK. Subsequently, we constructed a network of Traditional Chinese Medicine monomers targeting proteins through the HERB database. The molecules collected by HERB were MFAP5 and INHBA. MFAP5 was targeted by mercury, while INHBA was targeted by glycerin, citric acid, 17-beta-estradiol, and 2-butenal (Figure 7D).

### Experimental validation of INHBA expression and its correlation with M1 macrophages

**Figure 8:**
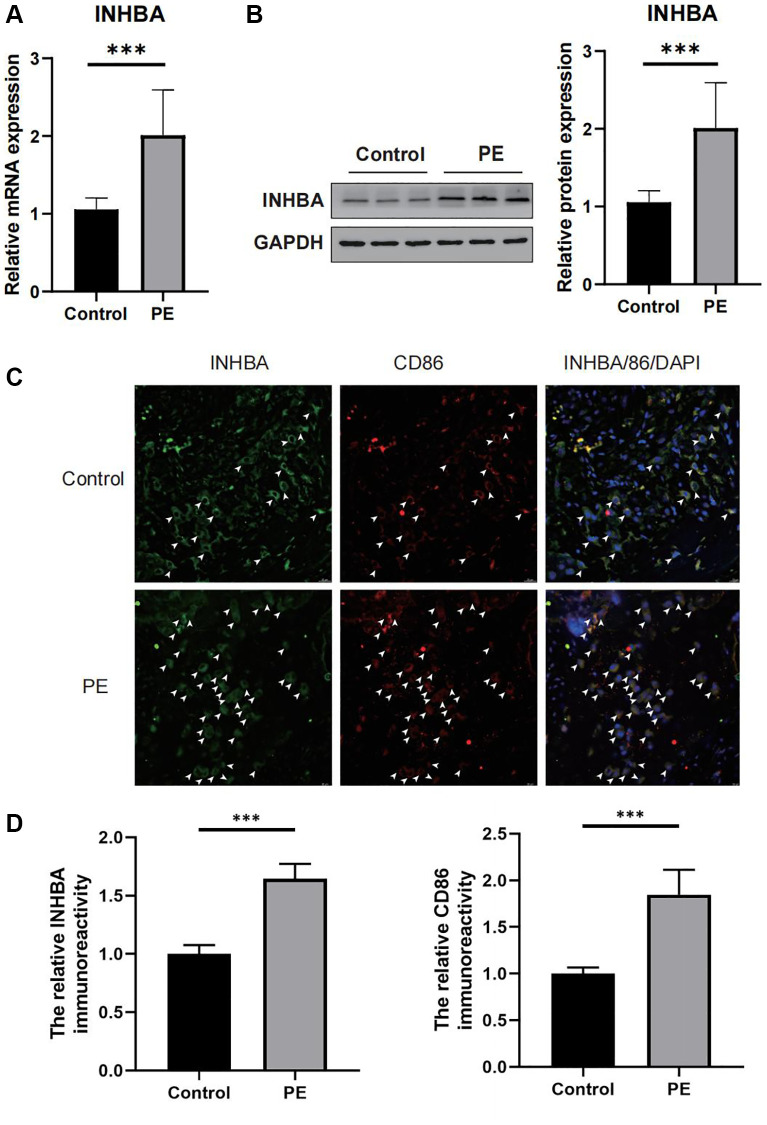
**Verifying the abnormal expression of INHBA in preeclampsia (PE) tissues.** (**A**) qPCR results for the expression of INHBA in both PE and control tissues. ^***^P < 0.001. *n* = 10. (**B**) Western blot results for the expression of INHBA in both PE and control tissues. ^***^P < 0.001. *n* = 10. (**C**) Representative immunofluorescent co-localization images of M1 macrophages with INHBA. White arrows indicated positive stained signals. (**D**) Quantitative analysis results for the relative immunoreactivity of placental INHBA and CD86 in all groups (3 sections for each sample). Scale bar = 20 μm. ^***^P < 0.001. *n* = 3.

The qPCR outcomes demonstrated that the expression of INHBA was noticeably higher in the placental tissues of PE patients when compared to normal placentas (as illustrated in Figure 8A). Similarly, Western blot analysis revealed an elevated protein expression of INHBA in the placental tissues of PE patients than in regular tissues (as shown in Figure 8B). Through the utilization of immunofluorescence staining, we assessed the correlation between INHBA and M1 macrophages. The results indicated a significant increase in both M1 macrophage quantity and INHBA content in the placental tissues of PE patients compared to normal placentas (as depicted in Figure 8C, 8D). Further co-localization analysis demonstrated the presence of merged signals for INHBA and M1 macrophages in the placental tissues of PE patients (as shown in Figure 8C, 8D).

## DISCUSSION

The placenta is a vital tissue that plays an essential role in supporting fetal growth. It regulates nutrient transportation, gas exchange, and hormone production during pregnancy, all of which are necessary for the survival of the fetus. The malfunctioning of the placenta can lead to many diseases, including PE, preterm birth, fetal growth restriction, and gestational diabetes [14]. PE is a distinctive complication of pregnancy that manifests after the 20th week of gestation. Its identifying features are the onset of hypertension and proteinuria or other signs/symptoms without proteinuria. If the condition progresses, it may cause severe complications such as cerebral hemorrhage, eclampsia, renal failure, disseminated intravascular coagulation, pulmonary abscesses, and even maternal and infant death [15, 16]. However, many patients with hypertensive disorders of pregnancy may not display any clinical symptoms during the early stages. Thus, PE can only be reliably detected when the symptoms appear in the second half of pregnancy, by which time damage to both mother and baby has likely already occurred. Therefore, accurate prediction, diagnosis, and timely treatment of this disease are crucial [17].

The changes in placental proteome that occur during PE are unclear. We attempted to obtain a comprehensive protein profile of differentially expressed placental proteins from PE placentas and to integrate the corresponding gene expression profile with proteome analysis to reveal key genes leading to PE pathogenesis. The findings provided new insights for potential biomarkers of PE prognosis and treatment strategies. We performed a comparative analysis of proteome profiles of placental tissues from PE patients and normal pregnant individuals. There were 216 downregulated and 106 upregulated proteins identified in the PE group compared with the normal group. Gene expression analysis was performed using microarray data of PE samples downloaded from public databases revealed 864 upregulated and 727 downregulated mRNAs in the PE samples compared with normal samples. An enrichment analysis was applied to investigate the proteins that were differentially expressed between the PE and control tissues. These proteins were mainly associated with the immune system, embryonic development, and immune cells. Pregnancy is an immune process in which the immune system should allow fetal growth and protect the mother and fetus from pathogen invasion. Although PE is a vascular disease, immune mechanisms are also involved [18]. Immune system changes are widely recognized as crucial determinants of PE. Building and maintaining a balance of maternal and fetal immunity is a prerequisite for normal pregnancy. PE, as a systemic inflammatory response, leads to an imbalance between placental material and maternal adaptation [19]. Immune cell infiltration is a novel bioinformatics technique that has been used to study diagnostic and prognostic markers for various diseases. Macroscopic quantities of macrophages have been found to differ between PE patients and normal pregnant individuals, and their polarization state also differs. The total number of macrophages in the placenta of PE patients increases, while the numbers of M1 and M2 macrophages increase and decrease, respectively, compared with normal pregnant individuals [20]. In this study, we found that M1 macrophages were significantly upregulated and M2 macrophages were significantly downregulated in placental tissues of PE patients through immune cell infiltration analysis, consistent with previous studies.

To investigate mRNA related to M1 macrophage infiltration in PE, we employed WGCNA. This is a systemic biological method that helps to identify highly correlated gene modules and gene sets in order to establish links between gene sets and phenotypes, thus identifying potential therapeutic targets or biomarker genes. WGCNA delivers information regarding biological pathways and has emerged as a valuable tool in many medical fields [21]. We identified three positively correlated modules and two negatively correlated modules related to M1 macrophage infiltration, containing a total of 378 mRNAs, which were included in subsequent analyses. In order to identify proteins related to M1 macrophage infiltration, we intersected the differentially expressed proteins and mRNAs, and M1 macrophage-associated mRNAs. Four proteins were identified: HTRA4, POGK, MFAP5, and INHBA. We confirmed them as new candidate biomarkers of PE, and immune cell infiltration analysis confirmed that the transcriptional expression levels of HTRA4, POGK, MFAP5, and INHBA were positively correlated with M1 macrophage infiltration scores and negatively correlated with M2 macrophages, B cells, monocytes, dendritic cells, neutrophils, NK cells, CD8+ T cells, and Treg cell infiltration scores. The characteristic of molecular networks is their ability to display complex interactions, including the regulation of gene expression and cellular function, and thus play a crucial role in the development and progression of diseases [22].

To further explore the function of hub proteins, PPI network construction and functional enrichment analysis were performed. The findings revealed that the proteins were related to cell differentiation, embryonic development, and multicellular biology processes. Pathway enrichment analysis showed that these proteins were related to signaling pathways, such as TGF-β, cell-cell cytokine receptor interaction, regulation of stem cell pluripotency, elastin-related molecules, and extracellular matrix organization. Previous studies have reported differential expression levels of TGF-β associated with PE patients and pointed-out that TGF-β played a key role in the development of PE [23, 24]. As well, cytokines are also involved in the invasion, differentiation, and angiogenesis of the trophoblast layer, which are the hallmark features of PE. M1 macrophages specifically phagocytose, produce reactive oxygen species, and secrete inflammatory cytokines in response to ligand binding, such as the binding of lipopolysaccharide to extracellular Toll-like receptors, while M2 macrophages produce anti-inflammatory cytokines, such as interleukin (IL)-10, IL-4, and Arginase 1, which exhibit immunosuppressive characteristics and participate in biological activities that include tolerance and tissue remodeling [21]. Disruption of the extracellular matrix may lead to interruptions of cell proliferation and invasion, failure of cell death, and loss of cell differentiation [25].

The foregoing core genes identified in our study have been reported in the literature regarding their related functional roles, particularly INHBA as a candidate serum marker for PE. HTRA4 is a placenta-specific serine proteinase that gradually increases in serum level around gestational week 24 to 25 during normal pregnancy and then remains relatively stable throughout the remainder of pregnancy. In early-onset PE, expression of HTRA4 in the placenta and circulating levels of HTRA4 are significantly increased at disease onset [26]. In addition, the increased placental-derived HTRA4 in plasma circulation is a potential cause of endothelial dysfunction, altering a series of endothelial genes related to inflammation, vascular activity, angiogenesis, cell adhesion, platelet activation, and coagulation [27]. POGK contains a transposase domain at the C-terminus and a KRAB domain at the N-terminus. Currently, the only reported study on POGK gene indicates its upregulation in hepatocellular carcinoma patients and correlates with poor prognosis and immune cell abundance in the tumor microenvironment, suggesting it as an effective therapeutic target for this patient population. However, the function of the protein encoded by the human POGK gene is still unclear [28]. MFAP5 has been closely related to the progression of gynecological diseases, such as breast cancer, ovarian cancer, cervical cancer, and uterine tumors. However, there is no report on the role of MFAP5 in PE [29–32]. Recent studies suggest that impaired remodeling of uterine spiral arteries due to inadequate infiltration of the maternal decidua and myometrium could be critical in the early stages of the development of PE. Upregulation of INHBA promotes human trophoblast invasion and plays a crucial role in early embryonic implantation [33]. Activin A, encoded by INHBA, is a homodimeric protein belonging to the TGF-β superfamily. During pregnancy, the placenta is the main source of circulating Activin A. In women with PE, maternal levels of Activin A are approximately 10 times higher than normal [34, 35]. Activin A levels may increase several months before the onset of preeclampsia [36]. Additionally, Activin A levels can predict post-PE cardiac dysfunction and may be helpful in monitoring the risk of remote cerebrovascular disease [37]. In the present study, qRT-PCR and Western blot analyses revealed that mRNA and protein expression levels of INHBA were significantly upregulated in preeclamptic placental tissue compared to normal placental tissue. Immunofluorescence co-localization experiments confirmed a significant positive correlation between INHBA and M1 macrophage recruitment and infiltration. Therefore, based on our results, INHBA is likely to play an important role in PE, at least as a key gene in immune cell infiltration. Increased expression of INHBA in the placenta may promote the development of PE by recruiting M1 macrophages to enhance trophoblast invasion capability, which deserves further investigation.

This study may provide new therapeutic targets for PE. By altering the expression levels of genes related to M1 macrophage polarization, it may be possible to reduce M1 macrophage polarization and increase M2 macrophage polarization, potentially improving the prognosis of patients with PE. However, our study also has some limitations. First, the potential PE-related biomarkers identified in this study require further literature support and laboratory evidence, such as animal experiments, for validation. Second, the sample size is relatively small. Studies with more samples are needed to further validate HTRA4, POGK, MFAP5, and INHBA as biomarkers for PE.

## CONCLUSION

The mechanism of PE was related to macrophage polarization. Proteins related to M1 macrophage polarization may serve as potential early diagnostic biomarkers for PE. HTRA4, POGK, MFAP5, and INHBA may be potential therapeutic targets for PE-related to M1 infiltration.

## Supplementary Materials

Supplementary Table 1

Supplementary Table 2
